# Imaging and radiation effects of gold nanoparticles in tumour cells

**DOI:** 10.1038/srep19442

**Published:** 2016-01-20

**Authors:** Harold N. McQuaid, Mark F. Muir, Laura E. Taggart, Stephen J. McMahon, Jonathan A. Coulter, Wendy B. Hyland, Suneil Jain, Karl T. Butterworth, Giuseppe Schettino, Kevin M. Prise, David G. Hirst, Stanley W. Botchway, Fred J. Currell

**Affiliations:** 1 Centre for Plasma Physics, School of Mathematics and Physics, Queen’s University Belfast, Belfast, BT7 1NN, Northern Ireland, UK; 2 Camlin Technologies Ltd. Lisburn, BT28 2EX, N.Ireland, UK; 3 Centre for Cancer Research and Cell Biology, Queen’s University Belfast, Belfast, BT9 7BL, Northern Ireland, UK; 4 Department of Radiation Oncology, Massachusetts General Hospital and Harvard Medical School, Boston MA, USA; 5 School of Pharmacy, Queen’s University Belfast, Belfast, BT9 7BL, Northern Ireland, UK; 6 Northern Ireland Cancer Centre, Belfast Health and Social Care Trust, Belfast, BT9 7AB, UK; 7 National Physical Laboratory, Hampton Road, Teddington, TW11 0LW, UK; 8 Central Laser Facility, Research Complex at Harwell, Science and Technology Facilities Council, Rutherford Appleton Laboratory, Oxfordshire, OX11 0QX, UK

## Abstract

Gold nanoparticle radiosensitization represents a novel technique in enhancement of ionising radiation dose and its effect on biological systems. Variation between theoretical predictions and experimental measurement is significant enough that the mechanism leading to an increase in cell killing and DNA damage is still not clear. We present the first experimental results that take into account both the measured biodistribution of gold nanoparticles at the cellular level and the range of the product electrons responsible for energy deposition. Combining synchrotron-generated monoenergetic X-rays, intracellular gold particle imaging and DNA damage assays, has enabled a DNA damage model to be generated that includes the production of intermediate electrons. We can therefore show for the first time good agreement between the prediction of biological outcomes from both the Local Effect Model and a DNA damage model with experimentally observed cell killing and DNA damage induction via the combination of X-rays and GNPs. However, the requirement of two distinct models as indicated by this mechanistic study, one for short-term DNA damage and another for cell survival, indicates that, at least for nanoparticle enhancement, it is not safe to equate the lethal lesions invoked in the local effect model with DNA damage events.

Physical approaches to the optimisation of radiation therapy increasingly require consideration of the biological factors involved in the targeting and killing of tumour cells. As with all forms of cancer treatment the aim of radiotherapy is to selectively maximise tumour killing while reducing the damage to healthy tissue. From the first demonstration of gold nanoparticles (GNPs) as a radiation contrast agent^1^, GNP radioenhancement/sensitisation has become an increasing area of investigation as an approach to increase the effectiveness of ionising radiation in biological systems. Gold’s general biocompatibility, combined with GNP’s apparent specificity to locate at tumor sites make GNPs an ideal therapeutic contrast agent. Consequent studies have shown the basis behind gold radiosensitization is due to gold’s inherent increased photoelectric absorption cross section in comparison to that of tissue^2^,^3^,^4^. During irradiation, this results in an enhancement of the energy deposition in the vicinity of the gold particles due to the generation of photoelectrons, Auger electrons, and characteristic X-rays^5^,^6^. Numerous theoretical studies have investigated the mechanisms behind the dose deposition as well as localisation of GNPs. However, while validating simple predictions of increased dose deposition, the enhanced physical and biological effects of GNPs measured experimentally both *in vitro* and *in vivo* greatly differ from those predicted by macroscopic models^7^,^8^,^9^. Instead, many studies report a significant difference between the concentrations of GNPs required experimentally to produce an observable effect and the much higher concentrations predicted theoretically.

In an attempt to further understand this disagreement, the Local Effect Model (LEM) was applied to this system to take into account the highly inhomogeneous dose distributions produced on the nanoscale following the introduction of a contrast agent. Originally developed to describe the effects of localised dose patterns found in charged particle therapy, the LEM was adapted to analyse the dose deposition produced by low energy secondary electrons surrounding the GNPs as opposed to the previous macroscopic view of calculating the average dose deposited over a much larger volume. While the LEM successfully demonstrates the relationship between the dose distribution inhomogeneity and observed cell inactivation in GNP enhanced radiotherapy^10^, the dominance of the short-range effects presented by this model further highlights the importance of GNP intracellular location. Monte Carlo simulation studies by McMahon *et al* and Carter *et al* have shown the dose deposited by secondary electrons escaping a GNP dropped significantly beyond 10 nm and 5 nm respectively from the particle surface^6^,^10^. Although this finding suggests that radiosensitisation can only occur if the GNPs are in close proximity to the nucleus (and DNA), significant radiosensitisation has been recorded *in vitro* for GNPs localised far from the cell nucleus^7^. Dose enhancement due to secondary electrons has been predicted as far away as 10 microns from the GNP surface^11^,^12^,^13^. As well as dependent on GNP size^8^,^14^, the level of enhancement is also cell line dependent further suggesting dose enhancement depends heavily on other biological factors, including localisation and distribution within the cells^15^,^16^,^17^.

All of the above observations emphasize the importance of improved understanding of the intracellular localisation and distribution of GNPs. The objective of this investigation was to compare the results of recently measured cell survival and DNA damage enhancements to those predicted by the LEM and other models. In addition, we have established a methodology to measure intracellular displacement between GNPs and nuclear DNA using multiphoton imaging, which enabled the production of a new biophysical model that more accurately predicts DNA damage through GNP radiosensitization. Both the incoming IR laser pulse and the outgoing UV photons are not obscured by either the cell or the GNPs as is evidenced by the technique’s ability to take Z-stacks throughout an entire cell. Furthermore, the UV photon detection is performed using a photomultiplier meaning that the measurement is undertaken in a pulse-counting regime governed by Poisson statistics. Hence, we regard this methodology as quantitative in the sense that it can give relative densities of GNPs throughout a cell.

## Results

The results from experimental measurements performed at the Diamond Light Source Synchrotron in Oxfordshire, UK are presented below. MDA-MB-231 cells were doped with 500 μg/ml of GNPs in media and left for 24 hours prior to irradiation. In this cell line, under these conditions there is typically a 14 fold increase in exposure concentration^18^ leading to approximately 0.7% wt/wt of gold. This is comparable with the levels of uptake achieved in tumours in mouse models^1^ suggesting the concentration used in this study is in the typical range for diagnostic and therapeutic applications. These cells were irradiated with varying incident photon energies from 10 keV to 60 keV. This energy range is far lower than the primary beam of photons usually used in external beam therapy. However, McMahon *et al.*^19^ have shown that the main interaction with gold nanoparticles for MeV primary beams is with lower energy shower particles in a similar energy range to the one studied here. Therefore this mechanistic study can elucidate relevant mechanisms. Furthermore, some superficial tumours (e.g. radioresistant melanoma) might benefit from lower energy primary beams.

**Figure 1:**
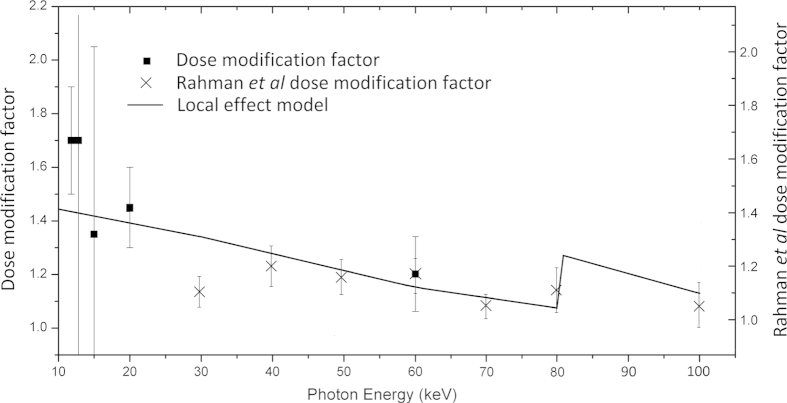
Graph showing relationship between experimental observations of clonogenic dose modification factor for increasing (monoenergetic) photon energy and predictions made using the local effect model for 20 nm gold nanoparticles (solid line). This works measured dose modification factor for MDA-231 cells containing 500 μg/ml of 1.9 nm AuroVist irradiated with 3 Gy (▀). Dose modification factor at 3 Gy for aortic endothelial cells containing 1.9 nm AuroVist at a mass concentration of 197 μg/ml (**X**)^35^.

Colony-forming assays were used to determine the long-term radiosensitising potential of GNPs. Figure 1 shows a good agreement between the comparison of the dose modification factor, calculated from the ratio of surviving fractions of clonogenic assay results, and the Local Effect Model (LEM) prediction, as described in the methods. Figure 1 combines experimental data from this work with previous results recorded under similar conditions by Rahman *et al.* the latter being scaled to create an equivalent dose modification value at 60 keV^18^. While direct comparisons between experimental data sets are not feasible, since different cell lines were used, both results follow the trend of the LEM for 20 nm GNPs with no maximum evident at 40 keV (as indicated by macroscopic dose enhancement calculations). It is interesting to note however, that a better numerical agreement was obtained using a 20 nm diameter for the LEM simulations rather than the 2 nm nominal diameter for the AuroVist nanoparticles. This is likely to be a consequence of nanoparticle-clumping in the cells, as is discussed below.

**Figure 2:**
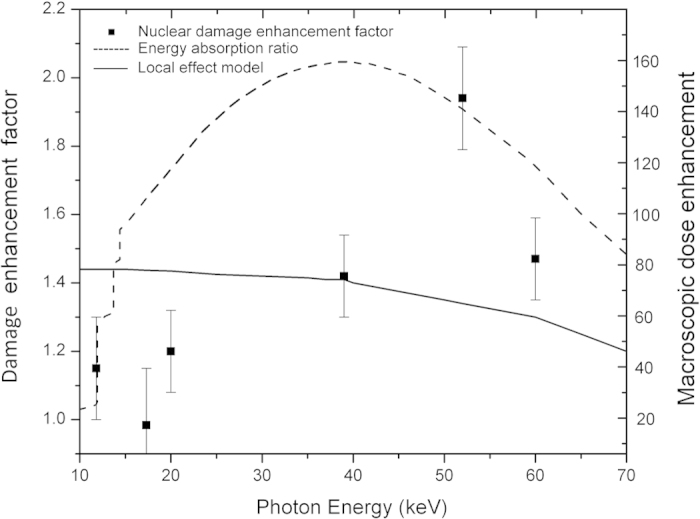
Measured nuclear DNA damage dose enhancement factor due to 1.9 nm AuroVist gold particles at a concentration of 500 μg/ml in MDA-MB-231 cells irradiated with 1.5 Gy for varying (monoenergetic) photon energy (squares). Cells were prepared as described in methods with DNA damage measured by counting γ-H2AX using immunofluorescent microscopy. Also shown is the dose enhancement factor for survival predicted by the local effect model (solid line) and the predicted macroscopic dose enhancement calculated from the energy absorption ratio of gold to soft tissue (dashed line).

Nuclei foci measured by γ-H2AX phosphorylation were counted 1 hour post irradiation. DNA damage enhancements, calculated from the ratio of nuclei foci for irradiated cells with GNPs, to cells without GNPs (background count subtracted), are shown in Fig. 2. Included in the figure is the predicted macroscopic dose enhancement as well as the dose modifying factor predicted for survival by the LEM^10^. Macroscopic dose enhancement is calculated from the ratio between the energy absorption coefficients of gold and soft tissue 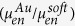. This is generally used as a prediction for the physical increase in the X-ray energy deposition.

Interestingly the energy dependence of the damage enhancement is inconsistent with both the macroscopic increase in energy absorption and the impact on clonogenic survival. Using the ratio of absorption coefficients, it is predicted that a significant increase in dose absorption above gold’s L edges at 11.8 keV to 14.5 keV may occur due to the increased ionisation associated with crossing the L-electron thresholds, whilst experimental results do not show a significant increase until higher energies.

These results, along with similar studies reported in literature^10^,^20^ indicate that while the LEM provides a good prediction of the long term clonogenic survival of cells doped with GNPs, the more immediate DNA damage induced a short time after irradiation under these conditions has a different behaviour. These results suggest a different form of interaction between the GNPs (or electrons emitted from them) and DNA molecules. Furthermore, at least for the case of GNP dose enhancement, these results indicate that short-term DNA damage is not a good predictor of cell survival and hence, the lethal lesions invoked in the LEM do not equate to DNA damage.

**Figure 3:**
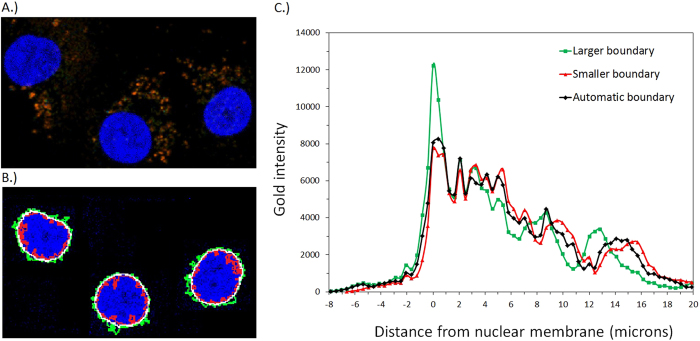
Co-registered image and analysis of 1.9 nm AuroVist gold nanoparticles in MDA-MB-231 cells. (**A**) Cells incubated with 500 μg/ml gold particles for 24 hours before being fixed and stained with DAPI. Image contains both the surface plasmon resonance signal from two-photon interaction with gold particles (orange) and nuclei regions (blue). The GNPs are observed in the cytoplasmic regions with some of the gold particles accumulating close to the nuclear boundary regions. (**B**) Nuclei boundaries produced in image analysis by a varying binary threshold. The automatic threshold determined by ImageJ (white) in comparison to a lower threshold (green) and higher threshold (red). (**C**) Integrated gold signal of 20 cells for increasing distance from the nuclear membrane for the three thresholds chosen in (**B**). The automatic threshold outline (black line) produces a maximum gold intensity at 0.5 μm outside the nuclear membrane. The gold intensity reduces significantly across the membrane (distance = 0) and the gold signal within the nucleus (distances < 0) is consistent with the assumption that all of the gold is outside the nucleus, instead reflecting the measured point spread function (PSF) for the gold image of 0.5 μm. The integration was done taking account of the number of pixels in the image at each distance from the nuclear membrane. Hence to a good approximation the intensity is equivalent to the relative density of gold nanoparticles.

Provision of information regarding the location of the GNPs relative to cell nucleus is available via the combination of two separate images; a GNP image obtained by means of a two-photon microscope exploiting the GNP’s plasmon resonance and a nuclear DNA image using a confocal immunofluorescent microscope. This process is explained in further detail in the methods section. The resulting image is shown in Fig. 3A.

**Figure 4:**
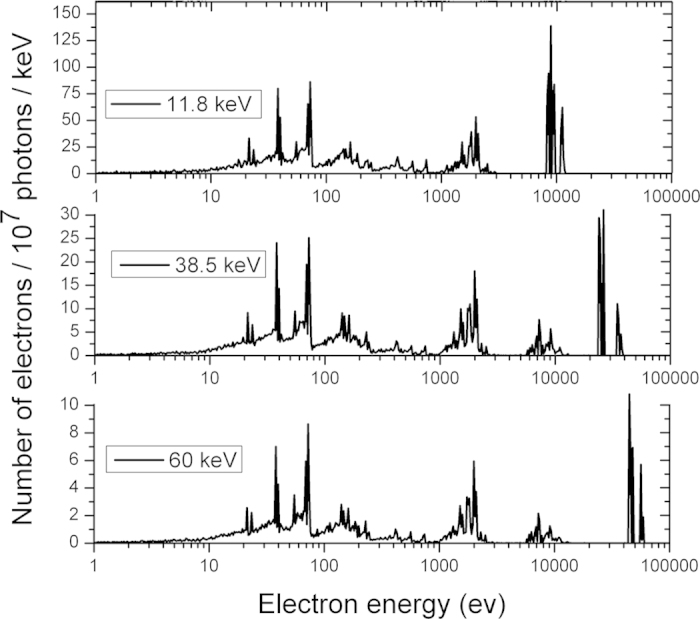
Geant 4 Monte Carlo simulations of the electron spectra produced after a 2 nm gold nanoparticle is ionised with 11.8 keV, 38.5 keV and 60 keV photons. Photon interaction with a GNP produces both the characteristic high energy photoelectron and a spectrum of lower energy Auger electrons resulting from an inner shell vacancy. The 2 keV and 10 keV Auger electrons contribution to the energy deposition is significant in the vicinity of the nanoparticle with ranges of around 100 nm and 1.5 μm in water respectively^25^.

The GNPs are found predominantly in the cytoplasmic regions of the cells with the majority of deposits accumulating close to the nucleus in the perinuclear protoplasm region as previously suggested with the aid of electron microscopy^18^. Co-registered nuclei and gold distribution images, similar to Fig. 3A, containing 20 cells were analysed by scoring the intensity of the gold signal for increasing distance from the nuclear membrane. The nuclear membrane location or nucleus-cytoplasm boundary was first determined in ImageJ^21^ software and the results illustrated in Fig. 3B. In calculating this boundary, 2 additional binary threshold values were used to enable an evaluation of the sensitivity of the conclusions reached regarding the final gold distribution to the threshold value automatically chosen by ImageJ. A lower threshold resulted in a larger boundary (green) in comparison to the automatic boundary (white), with the higher threshold resulting in a smaller boundary (red). The automatic threshold determined by ImageJ produced a nuclear boundary that followed closest to the DAPI image. Using this information the gold distribution with respect to the nuclear membrane was calculated and is shown in Fig. 3C. The distributions for each boundary followed a trend that was expected from the boundary size and position relative to the gold deposits. The plot shows that for the automatically chosen threshold (black line) the peak gold intensity occurs at 0.5 μm outside the nuclear membrane. The gold signal decreases significantly across the nuclear boundary to below 5% peak value within 1.6 μm inside the nuclear envelope. While the data presented here was obtained from two-dimensional images taken at the mid-plane of the nuclei, three-dimensional images of the cell and GNP distribution have also been produced. No definitive evidence was found in any of the images analysed to confirm the existence of considerable quantities of gold within the nuclei of MDA-MB-231 cells whereas the technique used would have been sensitive to any such component. The gold intensities with negative displacements shown in Fig. 3 are consistent with the measured point spread function (PSF) for the gold image of 0.5 μm. It is therefore possible to conclude that for MDA-MB-231 cells the most likely distribution for the GNPs places them exclusively outside the nucleus. Figure 3C shows the bulk of GNPs are at a distance equal to or greater than 0.5 μm from the nuclear membrane. Factoring in the nuclear envelope thickness, which has been estimated to be approximately 50 nm^22^,^23^,^24^, increases the attenuation of the electrons further. In fact, this factor is taken into account by our DAPI stain since it only highlights DNA and not the nuclear membrane. Evaluating the effects that the GNP distribution has on the nuclear DNA requires a more detailed inspection. Monte Carlo simulations that quantify the electron energies produced from the interaction of a range of photon energies with a gold particle are shown in Fig. 4.

Following an ionising event in a gold particle an energy range of electrons are produced. These include a (usually) relatively high energy photoelectron, succeeded by a range of Auger electrons all with energies ≤10 keV, that are emitted as a result of the Auger cascade that takes place as the inner shell vacancy left by the ionisation event is filled. The Auger electrons have energies of about 100 eV, 2 keV and 10 keV as is evident from the spectrum of secondary electrons shown in Fig. 4. The 100 eV electrons have a short range of around 50 nm, so deposit all of their energy near the nanoparticle surface. The higher energy electrons of 2 keV and 10 keV are more relevant to the proposed system due to their ability to deposit energy up to a distance of 100 nm and 1.5 μm in water respectively^25^. Auger electrons are therefore the dominant source of dose in the vicinity of the gold particle within several hundred nanometres of the GNP surface.

As mentioned above, while providing high doses in the vicinity of the nanoparticle, beyond several hundred nanometres from the gold particle surface Auger electrons are no longer the dominant source of dose enhancement. Nuclear DNA is consequently outside the range of the majority of the low energy Auger electrons, which play a significant role in LEM enhancement predictions. In agreement with this conclusion, the DNA damage enhancement for incident X-ray energies <20 keV is lower than predicted by absorption coefficients (Fig. 2) due to the proposed reduced effectiveness of low energy Auger and photoelectrons. The Auger spectrum that follows an ionisation event does not depend on the energy of the incident ionising particle, but rather on the shell from which the photoelectron was emitted. The Auger contribution therefore remains small and is little changed above 14.5 keV. Increasing photon energy however, increases the effectiveness of the photoelectron in dose deposition to the nucleus; a 50 keV electron can travel up to 20 μm in water^25^. This significantly increases the volume over which energy deposition can occur and hence the probability of photoelectron interaction with a DNA molecule within the nucleus.

**Figure 5:**
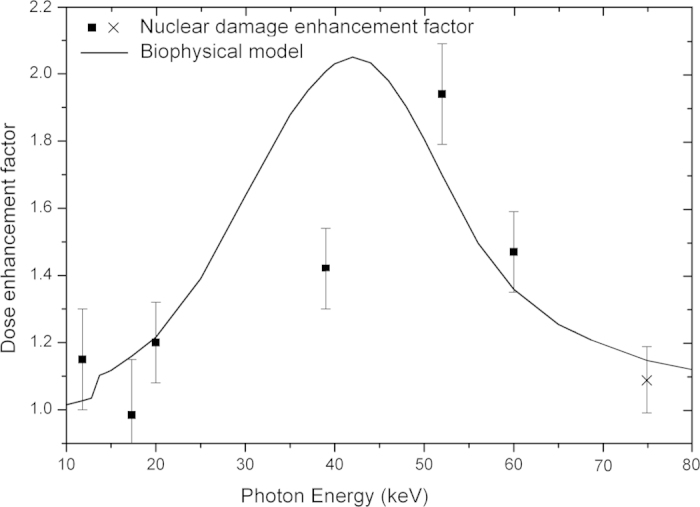
Experimental results and model prediction of the DNA damage enhancement factor for varying incident (monoenergetic) photon energy. Synchrotron results (squares) are shown alongside a measurement using a Faxitron 160-CP broadband X-ray energy cabinet source (cross). Also plotted is the predicted result from the biophysical model (line). The model result is expected to shift to higher energies, with the inclusion of various factors described in the main text.

A biophysical model has been developed that considers the volume over which the photoelectrons produced by an ionization event from the gold particles can cause DNA damage. The purpose of this model is not to capture all of the possible interactions in a complete manner but rather to account for the photoelectron’s role in a simple and physically transparent way (i.e. we avoid appealing to results from radiation track structure calculations). The model is described more fully in the methods section below, but briefly, dose is considered to be deposited in a sphere about each nanoparticle, with the radius of that sphere being equal to the maximum range of the photoelectron, this radius depending on the photoelectron energy and hence in turn on the photon energy. The enhancement to DNA damage caused by the photoelectrons is determined by considering the overlap of the cell nucleus and this sphere into which photoelectron dose is uniformly deposited, with integration over all initial GNP distributions being used to produce a final predicted DNA damage enhancement. Cell nucleus size and the GNP distribution are needed to predict the influence of the photoelectron. These were obtained from Fig. 3B,C respectively. The model’s predicted result for DNA damage enhancement is shown in Fig. 5 with reasonable (although not perfect) agreement to the experimental measurements. This prediction however is the result of the assumption that the gold distribution imaged in two-dimensions at the nuclei mid plane is uniform in three dimensions around the nuclei. Preliminary three-dimensional images have provided evidence that due to a variation in cytoplasm thickness a uniform gold distribution isn’t completely accurate, although it is a good approximation. Furthermore, the model ignores the effect of electrons emitted from GNPs from neighbouring cells and assumes dose is deposited uniformly inside the sphere of penetration. Inclusion of these effects into a more complete model is likely to shift the maximum predicted for the DNA enhancement to higher energy, bringing it more in line with the measurements. Nevertheless the broad agreement between the measured and modelled energy-dependence of the DNA enhancement suggests that the photoelectron is responsible for this damage. Included in Fig. 5 is a dose enhancement factor calculated using a Faxitron CP-160 X-ray cabinet source. The X-ray spectrum of this source is a broad, smoothly varying bremsstrahlung background ranging from 30 keV to 160 keV, with a mean X-ray energy of 75 keV^26^.

The model result confirms that in the case of GNPs with cell uptake following a similar distribution to the one shown in Fig. 3, short term (~1 hr) DNA damage is better represented by the effects of the high energy long range photoelectrons. With further development of the model to include the complete 3D gold distribution and taking into account the ability of one cell’s gold distribution interacting with a neighbour’s nucleus, we expect a shift of the 40 keV model peak to a higher energy. This is expected due to an increase in the average distance between nuclear DNA and the GNPs and is the subject of on-going research.

## Discussion

In previous studies there has been a significant discrepancy between theoretically predicted increases in X-ray energy deposition and experimentally observed increases in radiosensitization in the presence of heavy elements^8^,^15^,^27^. However the application of the LEM by McMahon *et al.*^10^,^19^ has provided a basis for the explanation of this increase in terms of the dose localisation near heavy atom nanoparticles^28^,^29^. In this work, the location of the gold particles in the cells and the structure of the energy deposition in the vicinity of particles for both clonogenic and DNA damage assays have been taken into account. It has been possible to relate both the LEM and a DNA damage model to two different biological outcomes, and show for the first time good agreement with experimentally observed cell killing and DNA damage production through the combination of X-rays and GNPs.

The local effect model predictions are in close agreement with the measured cell survival results of the clonogenic experiments. Smaller diameter nanoparticles should produce slightly more enhancement than predicted by the LEM for 20 nm GNPs presented, since for an equivalent mass uptake a greater number of low energy electrons will leave the nanoparticles at a given dose. The 20 nm results however may be a more effective estimate of the dose enhancement due to the aggregation of the gold particles in to clusters and the formation of biological vesicles^18^,^30^,^31^. The effectiveness of GNPs in long-term cell killing is driven by the Auger electrons and their characteristic inhomogeneous deposition of dose in the vicinity of the particles. We are therefore led to the conclusion that for high local doses there is little difference between the effects of applying localized doses to the nucleus in comparison to providing localized doses to the rest of the cell. Although this finding is somewhat at odds with the traditional view of radiobiology a growing body of evidence has emerged to suggest that nuclear DNA is by no means the exclusive radiation target in a cell^32^,^33^,^34^. Furthermore, whilst most radiobiology experiments are concerned with a dose range of 0–100 Gy the local doses found near the nanoparticles can be as high as 100,000 Gy^10^. Hence the finding applies to a new domain of radiobiology.

The experimentally measured short term (~1 hr) DNA damage is accurately predicted by a biophysical model using both the GNP location and the photoelectron as the main cause of damage. This work shows that short term DNA damage is driven by the photoelectron when gold particles are present in the perinuclear region. This finding is based on the observed physical distance that the large accumulations of gold particles are from the nucleus and the inherent thickness of the nucleus membrane (>50 nm). The paradox of increased cell killing with little evidence of the increase in short term DNA damage points to other mechanisms for cell killing at lower X-ray energies. The photoelectron can therefore be seen as playing a less effective role in cell killing, which is ultimately the most important endpoint when developing radioenhancers. It is important however to note that these findings do not exclude the development of longer-term DNA damage (i.e. with appearance kinetics peaked significantly after 1 hour) or lesions that are ultimately unrepaired, triggering cell death. However, such DNA damage would not be driven through either direct processes (i.e. electron mediated) or what are typically considered as indirect processes (i.e. radical formation via a primary interaction with water)^30^. This damage would instead be produced through the generation of much longer-lived species or biological processes. Investigations into the effects of irradiated GNPs on mitochondria and their subsequent effect on cell survival have been carried out^31^. The finding that GNPs induce both an oxidative cellular response involving reactive oxygen species along with mitochondrial depolarisation and oxidation support the hypothesis of a long term mechanism in GNP radiosensitisation.

Although this study is primarily of a mechanistic nature, it is interesting to speculate how these and associated results might be extended to more clinically relevant sources, with the caveat that a different nanoparticle might well show different sub-cellular localisation and hence drive somewhat different mechanisms. With regards to cell killing, these results provide further evidence for the applicability of the LEM and hence extension to clinical sources is as described by McMahon *et al.*^19^. However, with regards to DNA-damage related effects (e.g. induction of chromosome aberrations) the outcome would be somewhat different. For the extra-nuclear localisation shown here, only the relatively high-energy photo-electrons would contribute. The contribution could, in principle, be estimated by integrating the results of the biophysical model presented here over the shower spectrum calculated as described by McMahon *et al.*^19^.

## Methods

### Cell irradiation

Human breast cancer cell line, MDA-MB-231, was chosen due to its radiosensitisation in comparison to other cell lines^32^,^35^ when used with GNPs. Sample irradiation was carried out at the Diamond Light Source Synchrotron in Oxfordshire with two beamlines (B16 and I15) required to cover the desired incident photon energy range of 8 keV to 60 keV. Details of how the cells were maintained are given in Supplementary Information.

For DNA damage measurements, the cells were plated on cover slips and trapped between two silicon O-rings on a 16 well plate specifically designed for vertical mounting. This allowed the cells to remain in direct contact with media throughout the duration of irradiation. The plate was mounted in the vertical plane on an X-Y translating stage and a radiation raster pattern created by traversing the sample plate perpendicularly back and forth across a 2 mm × 2 mm X-ray beam. The cells were irradiated with 0 Gy, 0.5 Gy, 1.0 Gy and 1.5 Gy at set photon energies: 11.8 keV, 17.0 keV, 20.0 keV, 38.5 keV, 52.3 keV and 60 keV. Photon absorption was determined by measuring the photon fluence pre- and post-attachment of the sample plate and sample via a photodiode. Calculated attenuations due to the cover slip and sample plate were subtracted from the total absorption leaving a dose to sample value. After irradiation, cell plates were placed back in the incubator for 1 hour until fixing (see below). For the clonogenic assay the technique described by Puck and Marcus^36^ was used. Details are given in Supplementary Information.

### Imaging and damage enhancement calculation

Immunostaining was performed for the DNA damage marker γ-H2AX and detected using Alexa Fluor 488 secondary antibodies (Molecular Probes, UK). Cells were fixed one hour post irradiation, stained and then imaged using a confocal microscope. Details of the procedure are given in Supplementary Information. DNA damage enhancement values were calculated from the ratio of nuclei foci for cells exposed to gold and 1.5 Gy of radiation to cells with no gold and 1.5 Gy of radiation.

Imaging the GNPs was carried out using a two photon fluorescence lifetime imaging similar to the setup of Botchway *et al.*^34^. The experimental setup shared many of its parts with the confocal microscope used in acquiring the confocal images described above, apart from the excitation laser and detection method.

Due to the acquisition of cell and gold images on different setups of the same microscope a co-registration of the two images was required for both illustration purposes and post analysis, through the use of fiducial markers. Again more details are given in the Supplementary Information. All cell experiments were performed in accordance with the relevant guidelines and regulations, and approved by Queen’s University Belfast.

### LEM Enhancement

A brief description is presented in Supplementary Information whilst a more comprehensive descriptions and verification of the LEM can be found elsewhere^10^,^37^.

### Biophysical model

In constructing this model the emphasis was placed on simplicity to give a physically transparent explanation for the trend measured in Fig. 2. The model takes into account both the size of the cell nuclei and the mean displacement of the gold particle deposits from the nuclear boundary (Fig. 3). The nucleus was modelled as a sphere, with the GNPs considered to be homogenously distributed in a concentric spherical shell around the nucleus. An electron originating at any point within the gold distribution was considered to have equal probability of depositing energy anywhere inside a sphere, centred at the electron’s point of origin. The radius of the sphere was set to be equal to the penetration of the electron in water, i.e. the radius depends on the initial electron energy. In so doing, the effect of transport of the electron through the nanoparticle in which it was created is ignored. This is a reasonable approximation since the effect of the model (as argued in the main text above) is only seen with high energy electrons, which suffer negligible energy attenuation in transporting through this first nanoparticle. Also, for the subsequent transport the effect of other gold nanoparticles was ignored – this is reasonable because of the very low fractional density of gold used.

**Figure 6:**
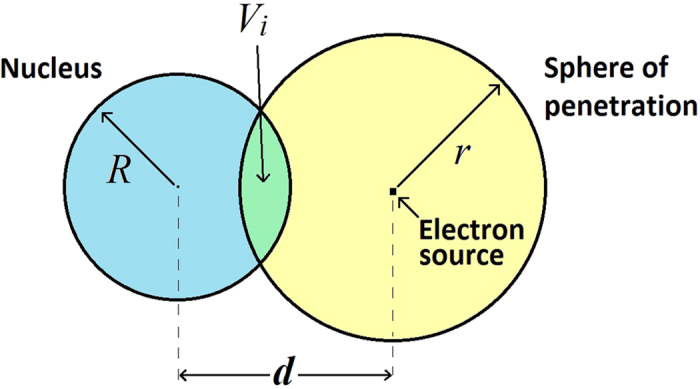
Schematic showing the radius of the nucleus *R*, the radius of the penetration of the electron generated at the gold particle *r*, the separation distance *d* between the nucleus centre and electron source and the volume of intersection *V*_*i*_ where a partial electron penetration volume overlaps the nucleus volume.

The total energy deposited inside the sphere is then equal to the initial energy of the electron to a very good approximation. The fraction of energy deposited in the nucleus is given by the ratio of the volume of intersection of two spheres, one representing the nucleus, the other the volume available for energy deposition of the electron. The total amount of DNA damage produced was considered to be proportional to the total amount of energy deposited in the nucleus. In the simplified model of a homogeneous distribution of GNPs around the cell nucleus with radius *R*, any particle of gold emitting an electron *e*, with energy *E*_*e*_ and penetration *r* can be represented by the following schematic in Fig. 6.

The energy deposited in the nucleus *E*_*D*_ is averaged over many such events and is equal to,


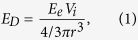


making the dose deposited to the nucleus proportional to,


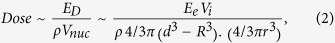


where ρ is the density of the nucleus treated as 1 g/cc. *V*_*i*_ can be evaluated analytically as a function of*r, R* and *d*. If 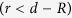 then *V*_*i*_ = 0 which represents electrons with energies which are too low to reach the nucleus. If 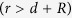 then 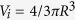 which represents high energy electrons with high penetration distances capable of passing right through the nucleus. Otherwise, 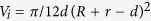

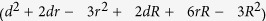
38. The dose due to the complete gold distribution obtained by averaging the dose deposited over the entire GNP distribution and is therefore proportional to,


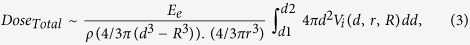


split into 3 branches, each of which can be done analytically. *d1* and *d2* represent the inner and outer boundaries of the homogenous distribution of gold particles. In our energy range of interest (1 keV to 100 keV) 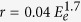 is derived from the log-log plot from Meesungnoen *et al.*^25^ where *r* is expressed in μm and *E*_*e*_ in keV. This value is in close agreement to the results of Dingfelder *et al.*^39^ of 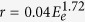. The electrons are created by photoelectric processes and are either Auger or photoelectrons. The enhancement due to ionisation from a given shell is therefore given by


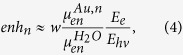


where *w* is the weight fraction i.e. weight of gold present/total weight, 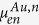 the mass energy attenuation of gold for a process *n* and 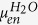 the mass energy attenuation for water at the photon energy *E*_*hv*_. For photoelectrons *E*_*e*_ = *E*_*hv*_ – *E*_*B*_ where *E*_*B*_ is the binding energy. Substituting this into equation (4) and combining with the dose enhancement in equation (3) we get,


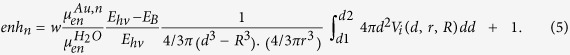


This function exhibits a maximum as a function of *E*_*hv*_ due to the 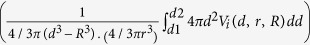 term. For a gold distribution predominantly localised near the nucleus this term initially grows as more electrons reach and interact with the nucleus, but it later falls off as the integral tends to a constant value while the 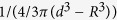 term continues to decrease. For the photoelectrons the term 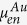 can be approximated with reference to the NIST database^36^ and the method for calculating the initial electron spectrum is explained in Supplementary Information.

The geometrical parameters for the biophysical model were derived from microscope images post co-registration, as described in Supplementary Information. An average nuclei radius of *R* = 7 μm was used in the model with a uniform gold distribution depth of 10 μm surrounding the nuclear membrane i.e. *d* = 7–17 μm.

## Additional Information

**How to cite this article**: McQuaid, H. N. *et al.* Imaging and radiation effects of gold nanoparticles in tumour cells. *Sci. Rep.* **6**, 19442; doi: 10.1038/srep19442 (2016).

**Data availability:** Supporting data are openly available at http://pure.qub.ac.uk/portal/en/datasets/search.html

## Supplementary Material

Supplementary Information
