# Supplementary material for: Imaging and radiation effects of gold nanoparticles in tumour cells
Source: Sci Rep. 2016 Jan 20;6:19442. doi: 10.1038/srep19442 (PMC4726169; doi:10.1038/srep19442)
Supplement: Supplementary Information [file srep19442-s1.pdf]

Imaging and radiation effects of gold nanoparticles in tumour cells

Harold N. McQuaid[1], Mark F. Muir[2], Laura E. Taggart[3], Stephen J. McMahon[3,4], Jonathan A. Coulter[5], Wendy B. Hyland[6], Suneil Jain[3,6], Karl T. Butterworth[3], Giuseppe Schettino[7], Kevin M. Prise[3], David G. Hirst[5], Stanley W. Botchway[8] & Fred J. Currell*[1].

Supplementary Information

Cell Incubation: Prior to irradiation, the MDA-MB-231 were maintained in Dulbecco's modified Eagle's medium (DMEM) supplemented with 10% foetal bovine serum and 1% penicillin/streptomycin. Cells were treated with 1.9 nm thiol capped AuroVist™ GNPs obtained from Nanoprobes Inc. (Yaphank, NY), diluted in media to a concentration of 500 μg/ml and incubated for 24 hours prior to irradiation.

Clonogenic Measurements: For the clonogenic measurements, cells were plated in multi-well plates containing culture medium supplemented with fetal bovine serum and 1% penicillin-streptomycin for 24 hours. The medium was removed and replaced with fresh medium containing 1.9 nm GNPs for a further 24 hours and irradiated at room temperature with 3 Gy for a range of photon energies. Following irradiation, cells were washed twice in phosphate-buffered saline, trypsinized, diluted four-fold to allow sufficient plating volume, vortexed at 1800 rpm for 10 seconds to achieve a single cell suspension, counted, and replated for survival analysis using the clonogenic assay technique described by Puck and Marcus[39]. Colonies were allowed to form following incubation at 37 ^{o}C, 5% CO_2, humidified for 14 days post irradiation before staining with 0.4% crystal violet and counted using a Colcount colony counter (Oxford Optronix). Plating efficiency was calculated as the ratio of colonies to cells seeded. The surviving fractions were calculated as the plating efficiency of the treated group divided by the plating efficiency of the untreated control cells.

Staining for Immunofluorescence: 1 hour post irradiation, cells we fixed using an equal parts acetone and methanol solution, permeabilized in an 0.1% solution of Triton X-100 in PBS and incubated with a mouse monoclonal primary antibody for SER 139 phosphorylation of H2AX. Cells were counterstained with 4,6-diamidino-2-phenylindole (DAPI) containing mounting medium for fluorescent microscopy (Vectorshield, UK). All images were obtained at the Central Laser Facilities Research Complex at Harwell where cell nuclei and foci were

viewed and counted via immunofluorescent microscopy on a Nikon eC1 confocal microscope with illumination provided by 405 nm and 488 nm excitation lasers respectively.

Imaging set up for nanoparticles: To image the nanoparticles, 600 nm laser light was obtained from an optical parametric oscillator pumped by a mode-locked Mira titanium sapphire laser producing 180 fs pulses. Custom made XY galvanometers were used for the scanning system and the laser beam was focused to a diffraction-limited spot through a water-immersion ultraviolet corrected objective. Surface plasmon emission was detected by a photomultiplier tube (Hamamatsu R3809U) and the characteristic decay lifetime recorded using a Becker and Hickl time correlated single-photon counting system (SPC830). Subsequent image analysis was performed using the SPCImage analysis software (Becker and Hickl, Germany). Due to the fast de-excitation of the gold surface plasmon (<2ns) a threshold on the signal lifetime within the fluorescence lifetime imaging microscope (FLIM) analysis software ensured that non correlating photons arriving at the detector were not included in the image analysis.

Image Co-registration: Fiducial markers were used consisting of 1 µm fluorescent beads soaked in 5-hydroxy tryptophan (5-HTP) and allowed to crystallize. The beads were detected on the confocal setup up through fluorescence provided by using the 488 nm laser and the two-photon excitation wavelength (600 nm) provides the excitation of the 5-HTP maxima at around 280 nm[35]. Two corresponding beads were chosen at opposite corners of the images and their relative positions used for image alignment. Only the two-photon image was manipulated during the co-registration process due to higher quality of the confocal image. Firstly the relative angle between the two beads in both the MP and confocal image were calculated and the MP image rotated to match the confocal. Following this procedure the first MP beads coordinates were shifted in X-Y to overlap the first confocal beads coordinates. The second beads MP coordinates were then stretched in X-Y to overlap its corresponding confocal bead. Cropping was required to produce a final image that contains information of both images in all regions. A visual check was carried out over the complete image and other images containing signal from multiple beads to verify accurate co-registration. These rotation, shift and stretch factors were then applied to the cell and gold images. The point spread function was measured from the 1 µm bead calibration image using the MosaicSuite plugin for ImageJ[21]. The plugin requires the numerical aperture and magnification factor of the microscope objective along with the camera pixel size and wavelength of the light detected.

A Note about the LEM: Instead of considering the cell-killing effects of an average macroscopic dose, the LEM considers the probability of damage occurring at each point in a cell based on the dose at that point alone, and calculates a surviving fraction based on the sum probability of damage occurring over the whole cell. While the LEM is typically applied to heavy ion therapies, it can be applied to any system where the nanoscale dose distribution can be calculated, such as the GNP-radiation interaction described in this work. To calculate the LEM-predicted survival for a given system, a radial dose distribution was generated in Geant4 for the corresponding combination of GNP size and incident X-ray energy. This provided values for the short-range dose contribution that results from an ionising event due to a GNP, and would be predicted to be highly inhomogeneous with dose. The GNP density, gold ionisation rate at the photon energy, and the total dose deposited were used to scale the volume affected by a single ionising event to describe the fraction of the total volume in which the short range dose is deposited. This dose distribution was then added to the effectively uniform background dose level which is delivered by X-rays that do not interact with GNPs to give the full dose volume distribution for a given combination of X-ray energy and GNP size.

This dose distribution was then used to estimate by survival by calculating the probability of a 'lethal lesion' occurring at each point within the cell based on the local dose. These lesions were then summed across the whole cell volume to give a cell survival probability, rather than calculating survival based on the average dose to the cell, which is typically done.

Determination of Geometrical Parameters for the Biophysical Model: The initial input parameters (*d,R*) for the biophysical model were calculated from microscope images post co-registration, showing both the nuclei regions and cell gold uptake. The processes used a combination of the image processing program ImageJ and a piece of analytical code written in the C programming language and are described as follows. Dapi stained nuclei images were first converted to binary images using ImageJ's 'Threshold' command. The 'Analyse Particles' command was then used on the binary image and the minimum pixel area increased to eliminate none nuclei artefacts. Selecting 'Bare Outlines' resulted in an image containing a boundary between the two regions of the binary image, and hence producing an estimated nucleus/cytoplasm boundary for the imaged cell nuclei. The initial threshold chosen by ImageJ directly influences the size of the nuclear boundary and this automatic result was compared against the boundaries produced by a manually increased and decreased threshold.

Threshold values ±10% or 5 intensity levels from the automatic value were used and all three shown in Figure 3b. This outline image was then processed in C where the centre of mass of each nuclei region was first calculated. From this position the distance to each nuclei boundary point was calculated via Pythagoras's theorem and the mean distance assigned the average nuclei radius. The nuclei outline coordinates were then read into a separate subroutine in parallel with the location and intensity of the gold particles in the multiphoton image. Selecting each gold pixel as a temporary origin the function searched radially outwards from this reference point to ascertain the nearest nuclear boundary. Gold signals that were found to be within the nuclei boundary were also included and given a negative displacement. This process was repeated for all gold signals to produce the complete gold distribution with the data presented using the nuclear membrane as the point of reference (Figure 3c).

Initial Electron Spectrum: Figure S1 shows the mass energy absorption of gold and also illustrates the method used for calculating the energy shell contribution. The energy mass absorption data can again be well approximated as straight lines on a log-log graph meaning a relationship of the form $\mu_{en}^{Au,n} = c_n E_{hv}^{m_n}$ can be used. Water energy mass absorption coefficients obtained from NIST also follow a straight line when in the energy range of interest for a log-log plot so can also be represented by $\mu_{en}^{H_2O} = c_{H_2O} E_{hv}^{m_{H_2O}}$. The binding energy threshold for the L-shell, E_B^L, is treated as 12.5 keV, below which only 3 keV or less Auger electrons are produced. As discussed above electrons with this energy are unable to penetrate the nuclear membrane. Above E_B^L for every photoelectron produced there is also a ≈ 10 keV Auger electron due to the L-M transition. This is included in the model and provides the small step increase observed at 13 keV in Figure 5. The contribution from all the lower energy Auger electrons however is negligible.

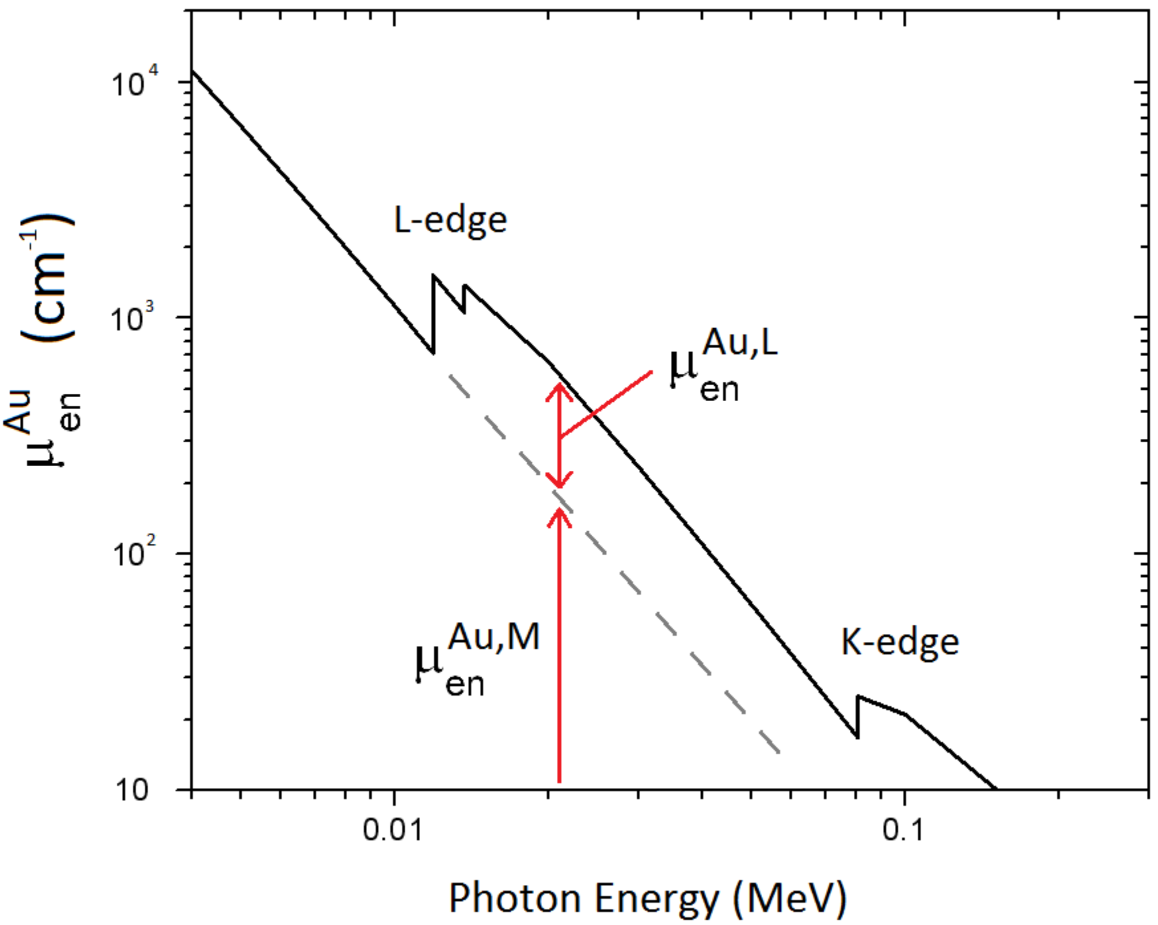

Figure S1. Plot illustrating the method used to calculate the L-shell contribution of the mass energy attenuation coefficient $\mu_{en}^{Au,L}$. Energy attenuation values for gold are obtained from the NIST database[39] and in the region of interest between shell edges attenuation values can be well approximated as straight lines on a log-log graph. The L-shell contribution to the total energy attenuation is found by extrapolating the M-shell values past the L-edge and subtracting this M-shell value from the total attenuation value.
